# Early antithrombotic therapy in patients with postinterventional cerebral hyperdensity reduces early neurological deterioration after mechanical thrombectomy

**DOI:** 10.1186/s12883-023-03497-9

**Published:** 2023-12-15

**Authors:** Yunhe Luo, Min Chu, Daosheng Wang, Xin Gu, Delong Wang, Jin Zheng, Jing Zhao

**Affiliations:** 1 Department of Neurology, Minhang Hospital, Fudan University, Shanghai, 201100 China; 2 Department of Neurosurgery, Minhang Hospital, Fudan University, Shanghai, China

**Keywords:** Postinterventional cerebral hyperdensity, Antithrombotic therapy, Early neurological deterioration, Acute ischemic Stroke, Mechanical thrombectomy

## Abstract

**Background:**

Initiation of early antithrombotic therapy after acute ischemic stroke (AIS) is crucial. We aimed to investigate whether early antithrombotic therapy influences early neurological deterioration (END) in AIS patients with postinterventional cerebral hyperdensity (PCHD) immediately after mechanical thrombectomy (MT).

**Methods:**

We retrospectively analyzed 108 consecutive anterior circulation AIS patients with PCHD immediately after MT. All patients were divided into END group and non-END group and END was defined as an increase of four points or more on the postinterventional National Institutes of Health Stroke Scale (NIHSS) score within the first 72 h after MT. Early antithrombotic therapy was defined as patients with PCHD who received antithrombotic therapy within 24 h after MT. Statistical analyses were performed to evaluate the association between early antithrombotic therapy and the risk of END.

**Results:**

Among 108 patients, 27 (25%) patients developed END. Multivariate regression analysis revealed that early use of antithrombotic therapy (OR = 0.229, 95%CI = 0.083–0.626, *P* = 0.004) was an independent protector of END and postinterventional low density shadow exceeding 1/3 of the vascular territory (OR = 4.000, 95%CI = 1.157–13.834, *P =* 0.029) was an independent risk factor for END.

**Conclusion:**

Antithrombotic therapy within 24 h after MT maybe associated with the reduced risk of END in anterior circulation AIS patients with PCHD.

## Introduction

Acute ischemic stroke (AIS) is a prevalent disease with high morbidity, disability, and mortality rates all around the world [1]. Currently, endovascular thrombectomy (MT) and thrombolysis are the most efficient treatments for AIS. But nearly 20% of patients who undergo MT will experience neurological deterioration within the first 72 h, partially as a result of vascular reocclusion [2]. According to a recent meta-analysis, 3–9% of AIS patients experienced vascular reocclusion within 24 h after MT, which is strongly associated with clinical deterioration and poor prognosis [3, 4]. Taking antithrombotic medicines as early as possible is crucial to prevent vascular reocclusion and thereby improve functional outcomes. However, it has been estimated that postinterventional cerebral hyperdensity (PCHD) may occur in up to 86% of AIS patients after MT [5]. Although 73–79% of them are contrast extravasation and only a small part is hemorrhagic transformation (HT) with no space-occupying effect, the use of antithrombotic drugs is still limited [6].

In general, the contrast extravasation and HT are distinguished by the high-density absorption of the non-contrast-enhanced computed tomography (CT) reassessment 24 h later. Although few hospitals have dual energy CT system that can tell the difference between HT and contrast extravasation, the positive predictive value of diagnostic accuracy of dual energy CT ranges from 33 to 100%, making it impossible to distinguish between the two accurately in most hospitals [7]. Although some doctors may prescribe antithrombotic treatments for PCHD patients, many are hesitant to do so for the few HT with no space-occupying effect in PCHD, which varies among doctors and hospitals [8].

Different pathophysiological pathways underlie HT and intracerebral hemorrhage [9, 10]. According to certain research, taking antithrombotic medications while having an asymptomatic HT will not result in more bleeding [11]. Moreover, a multicenter study also confirmed that early antiplatelet therapy is associated with a lower risk of END and a better neurological outcome in AIS patients when HT occurs after intravenous thrombolysis [12]. However, there has been no study to evaluate the efficacy and safety of using antithrombotic medications following PCHD. Consequently, many PCHD patients are unable to take antithrombotic medications within 24 h after MT due to the lack of evidence-based medical evidence.

In recent years, some PCHD patients in our center have received antithrombotic therapy within 24 h after MT. Therefore, this study aimed to investigate the association between early antithrombotic medication and early neurological deterioration (END) in anterior circulation AIS patients with PCHD after MT.

## Materials and methods

### Patients recruitment

This retrospective observational study was conducted in our hospital, a tertiary teaching hospital affiliated with Fudan University. We prospectively screened all AIS patients receiving MT from January 2019 to July 2022. The inclusion criteria were as follows: [1] aged 18 years or older; [2] patients with acute ischemic stroke caused by large vessel occlusion in the anterior circulation, which consist of internal carotid artery (ICA), M1 segment of middle cerebral artery, M2 segment of middle cerebral artery, and A1 segment of anterior cerebral artery; [3] performed C-arm computed tomography (CT) immediately following MT in the operating room; [4] high density on the brain CT immediately following MT which met the standard diagnosis criteria for PCHD. Patients were disqualified if they fulfilled the following requirements: [1] acute renal insufficiency, severe infectious fever, diabetic ketoacidosis, and acute heart failure that might exacerbate symptoms; [2] lack of available data. This study was reviewed and approved by the Institutional Ethics Committee of our hospital (ref. no. 2022-043-01 K).

### Data collection and imaging analysis

All data were collected prospectively. PCHD is defined as non-solid visually distinct parenchymal hyperdense areas found on the first postinterventional CT immediately following MT, with a surface area of at least 0.1 cm^3^, an increased density of at least 5 HU in comparison to the unaffected contralateral side, without a space-occupying effect, and adhering to the grey-white matter boundaries of typical anatomic structures(13). MT procedures included stent retrievers (SR) thrombectomy, contact aspiration (CA), or combined SR and CA. When the forward flow was difficult to maintain or the artery dissection was found during the operation, the treatment of stent implantation would be performed. For PCHD patients with successful reperfusion and without residual severe stenosis, the preoperative blood pressure was controlled to about 120–150/60–100 mmHg.

Antithrombotic therapy of PCHD patients following MT in this study was performed as follows. Considering the hyperdense without a space-occupying effect on the CT and the intraoperative heparinization, tirofiban (a maintenance dose of 0.2-0.3 mg/h for about 24 h without a loading dose and overlap with oral dual antiplatelet therapy for 4–6 h) was given only in the case of stent implantation and residual severe stenosis. For other atherosclerotic occlusion patients, oral dual antiplatelet therapy without a loading dose was given as soon as possible in 24 h. While for cardioembolic patients, oral antiplatelet therapy without a loading dose was performed (compared to dual antiplatelet therapy, single antiplatelet therapy was applied only to some cardioembolic patients with severe stroke symptom and low density on CT > 1/3 vascular territory after MT), and the time of initiating oral anticoagulant therapy (overlapping with low-molecular‐weight heparin in some cases, especially when warfarin was used) was determined by residual infarction volume and the stroke severity after MT. Oral antiplatelet agents were chosen from aspirin (100 mg/day), clopidogrel (75 mg/day), and cilostazol (100 mg twice daily, with a starting dose of 50 mg twice daily). Aspirin combined with clopidogrel is used as a conventional oral dual antiplatelet therapy. When aspirin or clopidogrel was contraindicated, cilostazol was prescribed as an alternative. When oral single antiplatelet therapy was chosen, the sequence of drug applications would be aspirin, clopidogrel, and cilostazol. Oral anticoagulant agents used in our department included warfarin (overlapping with low‐molecular‐weight heparin for at least 5 days and titrating with an INR range of 2–3), dabigatran (110 mg twice daily), and rivaroxaban (15 mg/day). Warfarin was usually used in patients with valvular atrial fibrillation, while either dabigatran or rivaroxaban was applied in patients with non-valvular atrial fibrillation. Low‐molecular‐weight heparin used in our department included enoxaparin (20–40 mg twice daily) and nadroparin (2050–4100 anti-Xa units twice daily). In particular, note that these antithrombotic strategies for PCHD patients were applied to some patients in 24 h, while others got antithrombotic medications after 24 h follow-up CT or even later CT re-examination. Because some attendings in our department were also worried about the safety and efficacy of early antithrombotic medication in AIS patients with PCHD.

Onset to reperfusion time (OTR), door to puncture time (DTP), and stroke subtype were evaluated by experienced neurologists. The Trial of Org 10,172 in Acute Stroke Treatment (TOAST) criteria were used to classify stroke subtypes. Stroke nurses gathered demographic and baseline information on patients with age, gender, smoking, diabetes, hypertension, and atrial fibrillation. In addition to baseline data such as stroke subtype and atrial fibrillation, postoperative stent implantation and residual stenosis were also collected to adjust for the effect of the relationship between antithrombotic and END.

A cranial non-contrast CT scan was usually conducted upon admission, immediately after MT, and during postoperative follow-up. Low density on immediate cranial non-contrast CT after MT was to assess the postinterventional obvious ischemia, and the TICI score was applied to the MT angiography to assess the degree of recanalization. The TICI score of 2b or 3 was considered successful recanalization. Massive cerebral infarction is defined as the low density shadow of head CT immediately after MT exceeding 1/3 of the vascular territory [13]. The initial occlusion site was evaluated by head and neck CT angiography before MT and confirmed by digital cerebral angiography of the occluded vessel. All the images were analyzed by two experienced neurologists who were blind to clinical features and study outcomes. In case of disagreement, images were reviewed until a consensus was reached.

### Clinical outcome

The National Institutes of Health Stroke Scale (NIHSS) was used to evaluate stroke severity before MT, immediately after MT, 72 h following MT, and whenever there is a deterioration in neurological function. END was defined as an increase of four points or more on the postinterventional NHISS score within the first 72 h after MT.

### Statistical analysis

Statistical analyses were performed using SPSS (version 26.0, IBM Corp, Armonk, NY, USA). All patients were divided into END group and non-END group. Normal-distribution continuous data were reported as mean ± SD and compared using the *t*-test, whereas non-normal-distribution continuous variables were expressed as median (interquartile range) and compared using the Mann-Whitney *U* test. Categorical variables were presented numerically (percentages, %) and compared between the groups using the relevant Fisher exact or Pearson *χ* 2 tests. A subsequent multivariate logistic regression analysis included the variables for which *P*<0.1 in the univariate analysis. *P*<0.05 (two-sided) was used to determine statistical significance.

## Results

### Clinical characteristics of patients

**Fig. 1:**
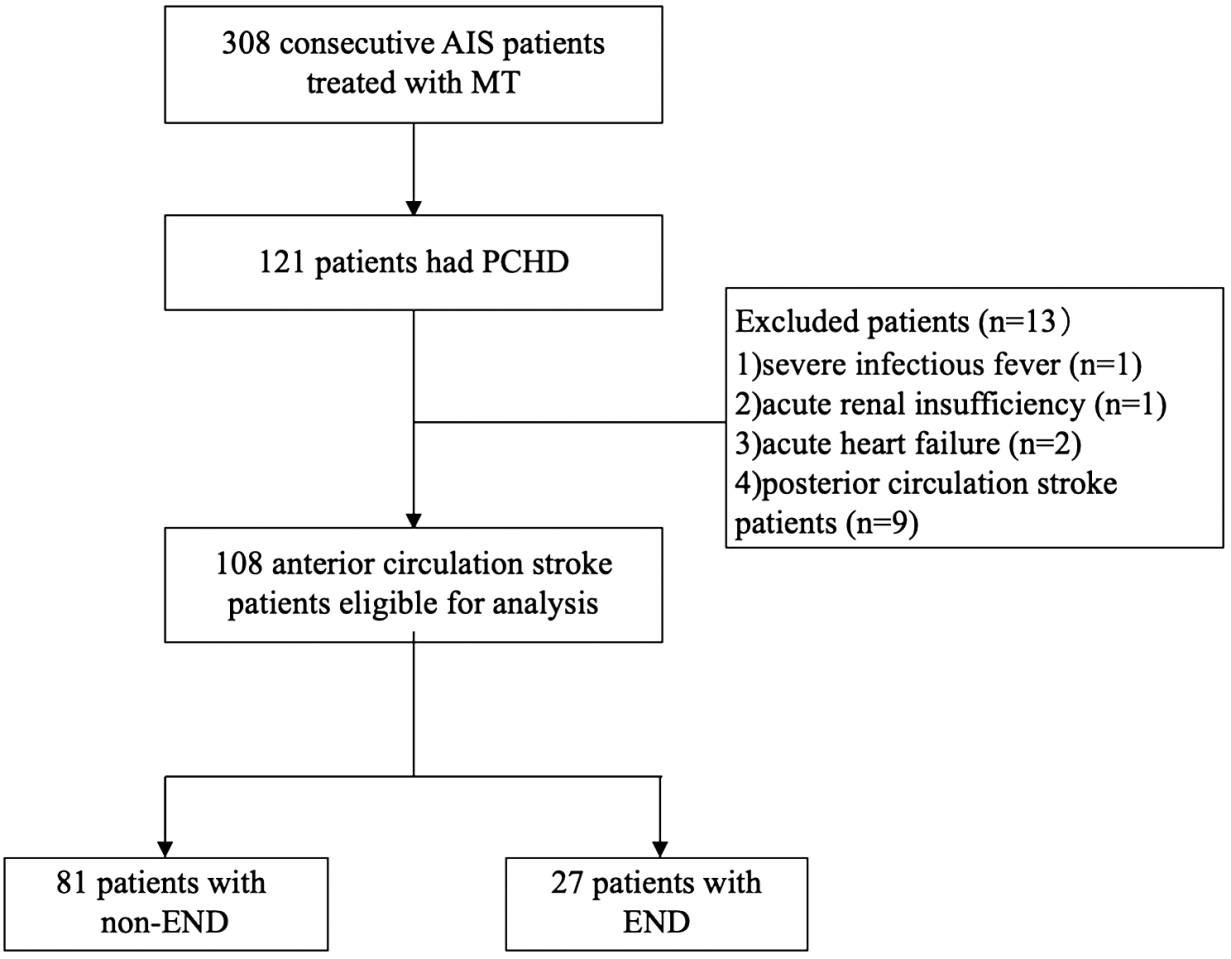
Diagram of the study recruitment

During the study period, we collected 308 consecutive AIS patients receiving MT. Among all the patients who received MT, PCHD occurred in 121 (39.3%) patients. After excluding patients with severe infectious fever (n = 1), acute renal insufficiency (n = 1), acute heart failure (n = 2), and posterior circulation stroke (n = 9), there were 108 patients included in this study. Figure 1 shows a diagram depicting study recruitment.

**Fig. 2:**
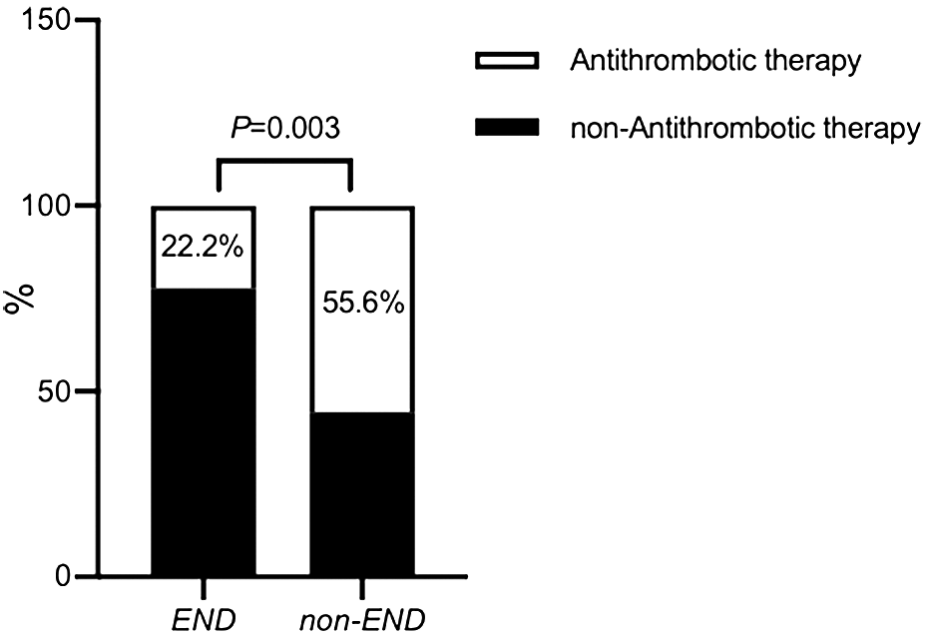
Antithrombotic therapy use in END and non-END groups

There were 73 (67.6%) men and 35 (32.4%) women, aged 25 to 96 (68.56 ± 14.32) years. Antithrombotic therapy was applied to 51 (47.2%) patients with PCHD within 24 h after MT. Vascular risk factors included diabetes mellitus (n = 63, 58.3%), atrial fibrillation (n = 52, 48.1%), hypertension (n = 65, 60.2%), and smoking (n = 23, 21.3%). All patients were categorized into two groups based on whether they had END or not. END occurred in 27 (25.0%) of the 108 patients. Patients in the END group had more portion of low density on CT > 1/3 vascular territory (33.3% vs. 11.1% *P* = 0.027) than those in the non-END group, and they showed more severe neurological deficits before MT (NHISS score 19.00 vs. 16.00, *P* = 0.014). Antithrombotic treatment was used less frequently in the END group than in the non-END group within 24 h after MT (22.2% vs. 55.6%, *P* = 0.003, illustrated Fig. 2). Demographic features and risk factors are summarized in Table 1.

**Table 1:** Baseline characteristics of PCHD patients according to the presence/absence of END

	END (n = 27)	non-END (n = 81)	*P* value
**Demographics**			
Age, median (IQR)	71.11 ± 15.72	67.70 ± 13.82	0.365
Men, n (%)	15 (55.6%)	58 (71.6%)	0.123
**Medical history**			
Hypertension, n (%)	15 (55.6%)	50 (61.7%)	0.570
Diabetes mellitus, n (%)	20 (74.1%)	43 (53.1%)	0.055
Atrial fibrillation, n (%)	16 (59.3%)	36 (44.4%)	0.182
**Current smoking, n (%)**	4 (14.8%)	19 (23.5%)	0.342
**NHISS score before MT**	19.00 (15.00–22.00)	16.00 (12.00–20.00)	0.014^*^
**OTR, median (IQR)**	325.00 (254.00-366.00)	294.00 (201.50-461.50)	0.731
**DTP, median (IQR)**	117.00 (75.00-152.00)	88.00 (78.00-125.50)	0.120
**Thrombolysis, n (%)**	12 (44.4%)	25 (30.9%)	0.198
**Stroke subtype, n (%)**			0.461
LAA	10 (37.0%)	42 (51.8%)	
CE	13 (48.1%)	31 (38.3%)	
SOE	1 (3.7%)	3 (3.7%)	
SUE	3 (11.1%)	5 (6.2%)	
**TICI, n (%)**			0.701
0	0 (0.0%)	2(2.5%)	
1	1 (3.7%)	2 (2.5%)	
2a	3 (11.1%)	5(6.2%)	
2b	8 (29.6%)	20 (24.7%)	
3	15 (55.6%)	52 (64.2%)	
**Low density on CT after MT, n (%)**			0.027^*^
Without low density on CT	7 (25.9%)	28 (34.6%)	
Low density on CT < 1/3 vascular territory	11 (40.7%)	44 (54.3%)	
Low density on CT > 1/3 vascular territory	9 (33.3%)^*^	9 (11.1%)	
**Residual vascular condition, n (%)**			0.723
Without stenosis	11 (40.7%)	29 (35.8%)	
Stenosis	16 (59.3%)	48 (59.3%)	
Occlusion	0 (0.0%)	4 (4.9%)	
**MT procedure, n (%)**			0.362
SR	0 (0.0%)	6 (7.4%)	
CA	0 (0.0%)	3 (3.7%)	
Combined SR and CA	27 (100%)	72 (81.9%)	
**Initial occlusion site, n (%)**			0.673
**ICA**	11 (40.7%)	30 (37.0%)	
**M1**	14 (51.9%)	42 (51.9%)	
**M2**	0 (0.0%)	5 (6.2%)	
**Tandem**	2 (7.4%)	4 (4.9%)	
**Stent implantation, n (%)**	3 (11.1%)	1 (1.2%)	0.078
**Antithrombotic therapy, n (%)**	6 (22.2%)	45 (55.6%)	0.003^*^

*means *P* < 0.05

*Abbreviations*: LAA, large-artery atherosclerosis; CE, cardioembolism; SOE, stroke of other determined etiology; SUE, stroke of undetermined etiology; SR, stent retriever; CA, contact aspiration; ICA, internal carotid artery

### Multivariate regression analysis of factors related to END

Variables with *P* < 0.1 in the univariate analysis were included in the multivariate logistic regression model. After adjusting for potential confounders(NIHSS scores before MT, diabetes mellitus, stent implantation, and low density on CT after MT), multivariate analysis revealed that the use of antithrombotic therapy within 24 h after MT (OR = 0.229, 95%CI = 0.083–0.626, *P* = 0.004) was an independent protector of END and low density shadow exceeding 1/3 of the vascular territory (OR = 4.000, 95%CI = 1.157–13.834, *P =* 0.029) was an independent risk factor for END (Table 2).

**Table 2:** Multivariate Regression Analysis of Factors Related to END

Variables	Univariate		Multivariate	
	OR (95%CI)	*P* Value	OR (95%CI)	*P* Value
Antithrombotic therapy	0.229 (0.083–0.626)	0.004	0.165 (0.046–0.591)	0.006
NIHSS scores before MT	1.084 (1.005–1.169)	0.036	1.028 (0.944–1.119)	0.523
Diabetes mellitus	0.396 (0.151–1.040)	0.060	0.478 (0.157–1.456)	0.194
Stent implantation	10.0(0.994-100.612)	0.051	13.319(0.613-289.552)	0.099
**Low density on CT after MT**	-	0.037	-	0.040
Without low density on CT	-		-	-
Low density < 1/3 vascular territory	1.000(0.347–2.885)	1.000	1.260 (0.381–4.164)	0.705
Low density > 1/3 vascular territory	4.000(1.157–13.834)	0.029	6.715 (1.432–31.482)	0.016

### Forest plot of odds ratios for END

**Fig. 3:**
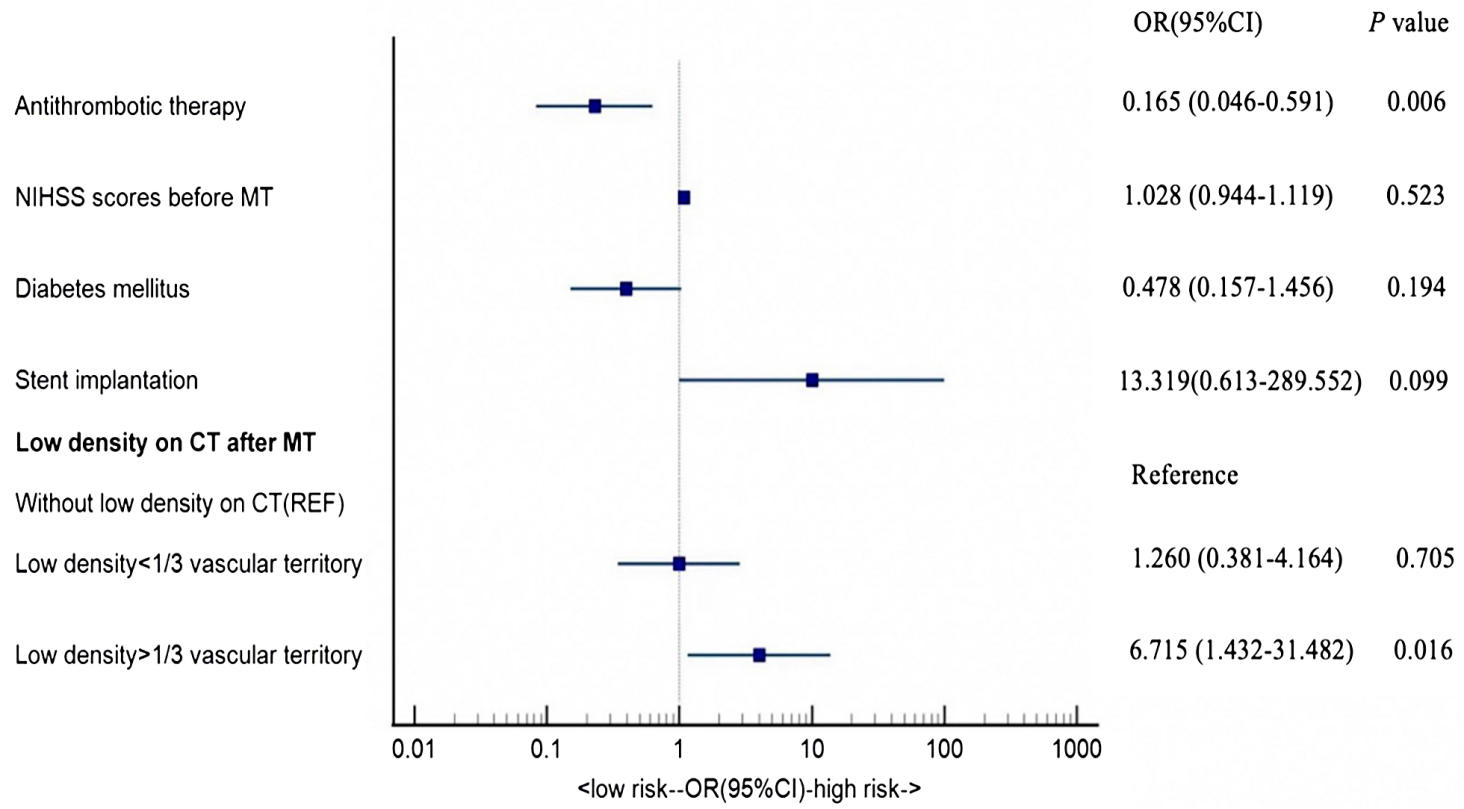
Forest plot of odds ratios for END

As shown in Fig. 3, forest plot revealed that low density > 1/3 vascular territory was a risk factor for END, whereas antithrombotic therapy was a protective factor (P < 0.05). Other indicators did not achieve statistical significance.

## Discussion

To the best of our knowledge, PCHD may be a meaningful concept which hasn’t been widely recognized, and our manuscript is the first study to investigate the relationship between early antithrombotic therapy and END in PCHD patients. Early antithrombotic therapy decreased the occurrence of END in patients with PCHD, and we also discovered that low CT density in more than one-third of the vascular territory following MT is an independent risk factor of END.

PCHD is a common complication following interventional stroke therapy [14]. The degree of PCHD corresponding to bleeding or contrast staining is unclear. When ischemia damage is restricted to the endothelial cell layer, PCHD is assumed to be contrast staining. However, PCHD may also be associated with hemorrhagic transformation when ischemia damage impairs the basal lamina [15, 16]. Although contrast extravasation occurs in 73–79% of PCHD, the remaining few HT without a space-occupying effect remains a major concern for antithrombotic therapy after MT [6]. Some studies have recommended distinguishing hemorrhage from contrast based on whether or not PCHD persists after 24 h or have advocated density criteria to predict (> 90 HU) or rule out (50 HU) underlying hemorrhage on follow-up CT [17, 18]. However, there are no accurate density thresholds on immediate postinterventional CT that make it possible to reliably distinguish between hemorrhage and contrast staining with acceptable specificity and sensitivity. In our study, the incidence of PCHD on immediate CT following MT was 39.3%, which was consistent with the incidence of PCHD reported by others (31.2–84.2%) [5, 19–21].

After the use of intravenous recombinant tissue plasminogen activator (rt-PA) and MT treatments, stroke care has shifted toward hyperacute stage management [22]. END is a prevalent and dreaded post-treatment consequence. Additionally, END is always associated with a poor three-month clinical prognosis, necessitating attentive and frequent monitoring of these stroke patients [23, 24]. The incidence of END in AIS ranges from 4.3 to 30% following MT therapy [2, 25, 26]. Our study demonstrates that the incidence of END was 25% after MT in anterior circulation stroke patients with PCHD. Furthermore, patients with PCHD who did not receive antithrombotic therapy had a 36.8% incidence of END, while those who did had a much lower 22.2% incidence. However, according to a recent research, 46.8% of patients with PCHD after MT experienced END [20]. The difference may be explained by the fact that antithrombotic therapy reduces the occurrence of END.

Many studies have demonstrated the use of antithrombotics in AIS patients can reduce the incidence of END. A previous observational study showed that taking aspirin when a stroke first occurred reduced the incidence of END by 97% [27]. A prospective cohort study also confirmed that pre-stroke aspirin use was independently associated with a lower incidence of END in AIS patients with atherothrombosis [28]. Moreover, It has also been proved that dual antiplatelet therapy is superior to single therapy in the early treatment of AIS [29]. Moreover, low-molecular-weight heparin (LMWH) therapy also dramatically decreased the incidence of END in patients with acute non-cardioembolic ischemic stroke, particularly those with large-artery stenosis [30]. For patients with AIS and large artery occlusive disease, treatment with LMWH within 48 h of stroke may reduce END during the first 10 days, mainly by preventing stroke progression [31]. Most recently, a multicenter study demonstrated that antiplatelet therapy did not increase the risk of hemorrhage expansion when HT was present [12]. These findings imply that antithrombotic therapy is beneficial in the treatment of ischemic stroke.

PCHD is thought to indicate varying degrees of ischemia damage to the blood-brain barrier (BBB), including contrast extravasation and HT [6, 32, 33]. The fundamental goal of a postinterventional CT is to rule out hemorrhage, therefore the presence of PCHD poses a diagnostic dilemma that may prevent the patient from receiving further treatment, such as the rapid initiation of antiplatelet therapy or the administration of anticoagulants [34, 35]. According to some studies, HT and intracerebral hemorrhage are caused by various pathophysiological mechanisms. Destruction of the blood-brain barrier is the pathological basis of hemorrhage transformation [36, 37]. Nevertheless, intracerebral hemorrhage is mostly caused by the localized dilatation or necrosis of the artery wall brought on by chronic hypertension, resulting in the formation of miliary microaneurysms and an increase in the fragility of the artery wall. When the blood pressure of a patient suddenly rises, the cerebral artery ruptures and bleeds [38]. Prior research has shown that early antiplatelet therapy is associated with a lower risk of END and a better neurological outcome in AIS patients when HT occurs after intravenous thrombolysis [12]. The 2018 Guidelines for the Early Management of Patients with Acute Ischemic Stroke also pointed out that the decision to initiate or continue antiplatelet or anticoagulant therapy can be made based on clinical evaluation results (IIB/-NR) [39]. Moreover, RESTART randomized trial found that antithrombotic therapy is beneficial for patients with a history of intracerebral hemorrhage [40]. Therefore, it makes sense that antithrombotic therapy can be safe and reduce END in PCHD patients following MT.

There are some strengths in our study. First, to the best of our knowledge, it is the first article that has specifically focused on whether antithrombotic therapy within 24 h in PCHD patients with anterior circulation stroke after MT could reduce the incidence of END. Second, to make sure that our results were as accurate as feasible, we did not include posterior circulation stroke and medical comorbidities that could lead to neurological deterioration in our analysis. However, our study also has some limitations that need to be considered. First, this analysis was performed retrospectively and without blinding, which has a chance of selection bias. Second, our study was single-center and limited in sample size, which means that despite rigorous adjustment for potentially confounding factors, we cannot eliminate the possibility of residual confounding in this observational study. And the findings should be validated in Large-scale prospective studies.

## Conclusion

Our study suggests that early antithrombotic therapy is associated with reduced risk of END in anterior circulation AIS patients with PCHD after MT. A further multicenter randomized controlled clinical trial is warranted to confirm our findings.

## Data Availability

The data that support the findings of this study are available from the corresponding author upon reasonable request.

## Abbreviations

AIS: Acute ischemic stroke
END: Early neurological deterioration
PCHD: Postinterventional cerebral hyperdensity
MT: Mechanical thrombectomy
NIHSS: National Institutes of Health Stroke Scale
HT: Hemorrhagic transformation
CT: Computed tomography
OTR: Onset to reperfusion time
DTP: Door to puncture time
TOAST: Trial of Org 10172 in Acute Stroke Treatment
